# The ability of chitosan–stearin as an edible coating on the quality of broiler chicken meat during cold storage

**DOI:** 10.5455/javar.2025.l876

**Published:** 2025-03-24

**Authors:** Yunilas Yunilas, Uswatun Hasanah, Trioso Purnawarman, Muheri Indra Aja Nasution

**Affiliations:** 1 Department of Animal Science, Faculty of Agriculture, Universitas Sumatera Utara, Medan, Indonesia.; 2 Division of Veterinary Public Health and Epidemiology, School of Veterinary Medicine and Biomedical Sciences, IPB University, Bogor, Indonesia.; 3 Department of Animal Production and Technology, Faculty of Animal Science, IPB University, Bogor, Indonesia.

**Keywords:** Broiler chicken, chitosan, cold storage, edible coating, meat, stearin

## Abstract

**Objective:**

This study aimed to evaluate how well fresh broiler meat may be preserved in cold storage using chitosan–stearin as an edible coating.

**Materials and Methods:**

A completely randomized design with a 3 x 5 factorial arrangement and three replications was employed. Factor I represented the formula dosage (FD) (FD0 = 0% chitosan + 0% stearin; FD1 = 1.5% chitosan + 1% stearin; FD2 = 3% chitosan + 1% stearin), while Factor II represented storage time (ST) (ST0 = 0 days; ST1 = 3 days; ST2 = 6 days; ST3 = 9 days; ST4 = 12 days).

**Results:**

The results showed that the water content, cooking loss, protein content, and fat content of broiler meat were significantly affected (*p *< 0.01) by the FD and ST. Nonetheless, no significant difference (p > 0.05) was observed in the meat’s ability to hold water. The broiler meat’s pH was significantly affected (*p *< 0.01) by the FD, but it was not significantly affected (*p *> 0.05) by the ST. Furthermore, no treatment underwent testing, which revealed the presence of *Escherichia coli *and *Salmonella* sp.

**Conclusion:**

Chitosan–stearin edible coatings with different formula doses FD and ST consistently preserve the quality of fresh broiler meat during cold storage, with average values of water content ranging from 48.97% to 53.73%, water-holding capacity from 17.52% to 34.30%, cooking loss from 10.03% to 33.19%, pH levels from 4.93 to 5.53, protein content from 14.54% to 17.46%, fat content from 20.55% to 24.21%, and no detectable presence of *E. coli* and *Salmonella* sp.

## Introduction

A well-liked source of animal protein due to its easy accessibility and reasonable price is broiler chicken meat. Nevertheless, fresh broiler meat has a relatively short shelf life because it tends to spoil easily during storage [1]. Alternative treatments have now been carried out in maintaining the quality of broiler meat by supplementing the feed during maintenance with the aim that before slaughtering broiler meat already has good quality and after slaughtering broiler meat will have a long shelf life, namely by giving supplements of organic acid origin [2]. Supplementation of grain origin such as wheat seeds in feed during the maintenance of broiler chickens has also been done [3]. In addition, supplements of starch origin, namely cassava, have also been carried out [4]. However, these alternative treatments are also not optimal, so the right handling in maintaining the quality of fresh broiler meat during storage is done by edible coating [5].

The application of edible coatings on broiler chickens has been widely carried out with various sources, one of which is the current trend, namely chitosan, which is the result of the deacetylation of chitin obtained by extraction [6]. Chitosan extraction involves several steps: demineralization, deproteination, depigmentation, and deacetylation. Chitosan dissolves in acetic acid, which is usually used at a concentration of 1%–2% [7]. Chitosan extraction will produce various yields and degree of deacetylation (DD) because of the various sources and concentrations of materials used in the extraction process and chitosan is also very multifunctional [8].

Edible coatings using chitosan that are currently trending have not yet used chitosan from crab (*Portunus pelagicus*) combined with palm stearin. Chitosan from crab (*P. pelagicus*) has a low mineral content from crab and shrimp shells of 22.93% and has a chitosan content of 20%–30% [9]. Chitosan also has the properties and characteristics of being biodegradable, antimicrobial, non-toxic, and a barrier to the escape of water vapor and gas in a product due to the polysaccharides of the strong chitosan coating [10]. In addition, stearin is a co-product obtained from palm oil by 20%–30%, which is still very limited in its use as an edible coating, in producing stearin through the stages of refining, bleaching, deodorizing, and cooling [11]. Stearin can be applied as a coating to improve water vapor permeability and flexibility, give a glossy finish, and maintain the product’s structure and shape during storage [12].

Research on the manufacture and use of crustacean-based chitosan has been conducted from shrimp shells and crab shell waste in various applications, including edible coatings. However, until now, no one has used a combination of chitosan from crab shell (*P. pelagicus*) waste and palm stearin applied to fresh broiler meat. Building on this premise, the authors conducted a study to test and evaluate the effectiveness of chitosan–stearin as an edible coating for maintaining the quality of fresh broiler meat during cold storage.

## Materials and Methods

The materials used in this study were chitosan of crab shell (*P. pelagicus*) degree of deacetylation (DD) 79.52% and palm stearin from the Bogor Palm Oil Research Center Unit, fresh broiler chicken meat from the traditional market, namely Pajak Pagi Padang Bulan Medan North Sumatra, 2% acetic acid, tween 80, Compact Dry EC, Compact Dry SL, gloves, alcohol, masks, distilled water, and label paper.

The tools utilized in this research include digital scales, filter paper, plastic, knives, cutting boards, basins, hot plates, stirrers, and rubber bands. This study employed an experimental method utilizing a completely randomized design with a 3 x 5 factorial pattern, including three replicates, totaling 45 research units. Factor I is the formula dosage (FD), namely FD0 = 0% chitosan + 0% stearin, FD1 = 1.5% chitosan + 1% stearin, FD2 = 3% chitosan + 1% stearin, and Factor II is the storage time (ST), namely ST0 = 0 days, ST1 = 3 days, ST2 = 6 days, ST3 = 9 days, ST4 = 12 days.

### Formula of edible coating

The preparation of the edible coating formula refers to Fahmi et al. [13]; chitosan from crab shell (*P. pelagicus*) is dissolved using 2% acetic acid at 40°C, while palm stearin is melted at 60°C. Then, the chitosan solution of the crab shell (*P. pelagicus*) was mixed with the palm stearin solution, and 2% tween 80 was added according to the variation of the ratio. Then, the solution was stirred for 4 min with a magnetic stirrer.

### Edible coating application

Fresh broiler meat that will be edible is coated and uses whole-breast parts that have been filleted (slicing using a knife with a thickness of 2 cm), and the need for fresh broiler meat used is 1.350 gm because each treatment uses 30 gm of fresh broiler chicken. To apply the edible coating to fresh broiler meat, the meat is dipped into the coating solution for 5 min and then allowed to dry for approximately 15 min until the coating adheres. The coated meat is then stored at 8°C.

### Variables

The variables measured were water content, water-holding capacity, cooking loss, pH, protein content, fat content, *E. coli,* and *Salmonella* sp.

### Data analysis

Data analysis was performed using SPSS software. Initially, variance analysis was conducted to determine the significance of the results. If the results were found to be significant, further analysis was carried out using the Duncan multiple range test.

## Results and Discussion

The results of the research related to physicochemical quality in the form of water content, water-holding capacity, cooking loss, pH, protein content, and fat content are shown in Table 1. According to this study, the average water content of broiler chicken flesh treated with edible coating ranged from 48.97% to 53.73% when exposed to a chitosan dose of up to 3% and stored for up to 12 days. The variance analysis results showed that the water content of broiler meat was significantly affected (*p *< 0.01) by the chitosan and stearin doses as well as the storage period, with no interaction between the dose and ST. While the FD0 therapy differed considerably from the FD1 treatment, it did not differ significantly from the FD2 treatment. This occurred because FD1 effectively reduced the rate of water vapor release from broiler meat, resulting in water content levels that were not significantly different from those observed with the FD0 treatment. The optimal water vapor permeability of broiler meat in this study occurred due to the combination of chitosan and stearin, which have their properties and characteristics in blocking the release of water vapor. In addition, the longer the broiler meat is stored, the water content will decrease due to the evaporation of water from the broiler meat. Storage of broiler meat coated with chitosan and stearin for up to 3 days was able to suppress the decrease in water content. However, along with the ST, there is a significant decrease in water content. According to Bhatia et al. [14], chitosan has the properties and characteristics to block the occurrence of water vapor. Basso et al. [15] stated that stearin also can inhibit water vapor. Pang et al. [16] added that changes in broiler meat water content are closely related to muscle protein because proteins in muscle have hydrophilic properties, namely the property of binding water molecules in meat.

**Table 1.** Physicochemical quality of edible coating broiler chicken meat.

	Water content (%)	Water-holding capacity (%)	Cooking loss (%)	pH	Protein content (%)	Fat content (%)
Storage Time (ST)						
0 day (ST0)	53.24^A^	22.21	12.74^B^	5.27	17.46^A^	24.17^A^
3 day (ST1)	53.11^A^	25.97	27.28^A^	5.04	17.32^A^	24.02^A^
6 day (ST2)	48.94^C^	26.92	26.83^A^	5.16	15.27^C^	22.05^C^
9 day (ST3)	49.46^B^	23.58	28.82^A^	5.19	16.01^B^	23.41^B^
12 day (ST4)	49.54^B^	27.83	27.67^A^	5.13	15.98^B^	22.31^B^
Formula Dosage (FD)						
0% (FD0)	50.61^B^	23.80	21.63^B^	5.35^B^	16.16^B^	23.45^A^
1.5% (FD1)	51.19^A^	24.19	26.75^A^	5.07^A^	16.72^A^	23.17^A^
3% (FD2)	50.78^B^	27.92	25.62^A^	5.06^A^	15.97^B^	22.01^B^
Interaction (ST x FD)						
ST0FD0	53.23	17.52	10.03	5.53	17.45	24.13
ST0FD1	53.73	22.21	12.41	5.00	17.21	24.21
ST0FD2	52.77	26.89	15.79	5.27	16.55	23.16
ST1FD0	52.93	23.56	25.50	5.20	16.21	23.05
ST1FD1	53.27	24.63	28.50	4.93	17.02	24.07
ST1FD2	53.13	29.73	27.83	5.00	17.14	23.98
ST2FD0	48.60	27.39	24.67	5.30	15.27	22.11
ST2FD1	49.20	27.53	28.83	5.13	16.05	23.84
ST2FD2	49.03	25.84	27.00	5.03	16.28	22.98
ST3FD0	48.97	26.11	23.64	5.43	16.28	22.76
ST3FD1	50.00	21.79	33.19	5.13	16.65	22.51
ST3FD2	49.40	22.83	29.63	5.00	15.02	21.27
ST4FD0	49.33	24.43	24.33	5.27	14.98	20.55
ST4FD1	49.73	24.77	30.83	5.13	14.76	21.01
ST4FD2	49.57	34.30	27.83	5.00	14.54	20.97

Means in a column with different superscripts differ at (*p *< 0.05).

The water-holding capacity of broiler meat treated with an edible coating in this study indicates that with a chitosan dose of up to 3% and an ST of up to 12 days, the average water-holding capacity ranged from 17.52% to 34.30%. The findings of the variance analysis showed that there was no interaction between the dose and ST and that neither the chitosan nor the stearin doses nor the ST had a significant effect (*p *> 0.05) on the broiler meat’s ability to hold water. Although the water-holding capacity of broiler meat did not show significant differences, it exhibited a numerical increasing trend. Furthermore, an edible coating with up to a 3% dosage was effective in preserving the water-holding capacity of broiler meat for up to 12 days of storage. This suggests that chitosan can safeguard protein myofibrils from damage (denaturation), thereby helping to maintain the water-holding capacity of broiler meat. According to Modzelewska-Kapitula and Zmijewski [17], muscle proteins’ capability to hold onto water is correlated with changes in water retention. Zhang et al. [18] stated that the higher the damaged myofibrillary protein, the percentage of water holding value decreases due to the large number of denatured proteins. Khalid et al. [19] added that the number of denatured proteins decreased the percentage of water-holding capacity value.

Cooking loss of broiler meat in this study revealed that with an edible coating treatment of up to 3% chitosan dose and ST of up to 12 days, the average cooking loss ranged from 10.03% to 33.19%. The findings of the variance analysis showed that the cooking loss of broiler meat was significantly influenced (*p *< 0.01) by the chitosan and stearin dosages as well as the ST, with no discernible interaction between the two variables. Compared to the FD1 and FD2 treatments, the FD0 therapy had a substantially lower level. This study’s broiler meat cooking loss stays within the normal (low) range, suggesting that cooking-related nutritional loss is comparatively little. According to Alfaifi et al. [20], meat that experiences low cooking loss is typically regarded as better than meat with high cooking loss because it preserves more nutrients when cooked. Latoch et al. [21] stated that meat with a low cooking loss is less likely to experience a significant loss of its nutritional content. Abril et al. [22] added that low cooking loss in meat will potentially reduce the nutritional content.

The pH value of broiler chicken meat with an edible coating treatment of up to 3% chitosan and an ST of up to 12 days ranged from 4.93 to 5.53 on average. The findings of the variance analysis showed that the pH of chicken flesh was significantly affected (*p *< 0.01) by the chitosan and stearin dosages. The pH of chicken meat was not significantly affected by ST (*p *> 0.05), and there was no interaction between the dose and ST. The higher the dose of edible coating chitosan, the lower the pH of the meat. The pH value of the meat decreased as the dose of chitosan increased. Edible coating with chitosan creates anaerobic conditions and facilitates the anaerobic glycolysis process, which leads to the production of lactic acid. The meat’s pH is lowered by the accumulation of lactic acid. In addition, the low pH value was due to the low water-holding capacity of the results. According to Olagunju and Nwachukwu [23], the reduction in meat pH is due to anaerobic glycolysis, which produces lactic acid. Rahman et al. [24] stated that the pH value is strongly linked to water-holding capacity; anaerobic glycolysis activity lowers the pH value, leading to a reduced water-holding capacity. Azad et al. [25] added that the pH value is closely connected to the presence of microbes in meat, thereby affecting its durability and quality.

The protein content of broiler meat has been edible coating in this study showed that edible coating treatment up to 3% chitosan dose and ST up to 12 days obtained an average range of 14.54%–17.46%. The variance analysis results showed that the protein content of broiler meat was significantly affected (*p *< 0.01) by the chitosan and stearin doses as well as the storage period, with no interaction between the dose and ST. The protein content of broiler meat with edible coating in this study decreased compared to before edible coating. This occurs because of protein degradation during storage, causing a decrease in broiler meat protein levels. This protein degradation greatly affects the decrease in protein levels that occur, but the decrease is not drastic. This study uses chitosan that has been dissolved using acetic acid so that the amino acid bonds are broken so that many protein bonds are dissolved. The use of acetic acid in dissolving chitosan also needs to be considered. In addition, the protein content of broiler chicken meat experiences protein denaturation during storage, which causes the protein side groups to open and protein solubility to decrease; the protein is separated. According to Katsumata et al. [26], along with the length of storage carried out, the protein will decrease due to protein degradation. Roman-Doval et al. [27] stated that chitosan that has been dissolved using acetic acid, which is acidic, will break down amino acids so that many protein bonds are dissolved. Ji et al. [28] added that denaturation will also occur in broiler meat proteins during storage, which causes the protein to decrease.

The fat content of broiler meat has been edible coating in this study showed that edible coating treatment up to 3% chitosan dose and ST up to 12 days obtained an average range of 20.55%–24.21%. The variance analysis results showed that the fat content of broiler meat was significantly affected (*p *< 0.01) by the chitosan and stearin doses as well as the storage period, with no interaction between the dose and ST. The fat content of broiler meat has been done; coating in this study decreased compared to before edible coating. This happened because this study used chitosan, which resulted in a decrease in fat. Chitosan has amino groups that cause chitosan to have high chemical reactivity as well, so chitosan has hydrophobic properties, which can cause fat (decrease in fat). In addition, damage to the quality of broiler meat can be caused by fat degradation during storage. According to Yaghoubi et al. [29], the use of chitosan in a product such as broiler meat during storage will experience a decrease in fat content. Ul-Islam et al. [30] stated that the decrease in broiler meat fat content occurs due to high chemical reactivity during storage, which can bind fat. Rukmini et al. [31] added that the decrease in fat in broiler meat during the storage period occurs due to the degradation process so that fat can decrease.

*Escherichia coli* is a bacterium that is prone to contaminating chicken meat [32]. Bacterial contamination of chicken meat typically originates from the room, equipment, and tables used for slaughtering, as well as the water utilized throughout the cutting and processing stages [33]. Meanwhile, *Salmonella* sp. is one of the pathogenic bacteria [34]. These bacteria can cause foodborne disease and become a big problem if not overcome, and *Salmonella* sp. is also believed to be a bacterium that causes salmonellosis and is zoonotic [35]. Meat that does not contain (negative) *E. coli* and *Salmonella* sp. will be safe and can be consumed. Warmate et al. [36] stated that food or meat that does not contain *E. coli* and *Salmonella* sp. will be safe and can be consumed.

**Table 2.** Microbiological quality of edible coating broiler chicken meat.

	*Escherichia coli*	*Salmonella* sp.
Storage Time (ST)		
0 day (ST0)	Negative	Negative
3 day (ST1)	Negative	Negative
6 day (ST2)	Negative	Negative
9 day (ST3)	Negative	Negative
12 day (ST4)	Negative	Negative
Formula Dosage (FD)		
0% (FD0)	Negative	Negative
1.5% (FD1)	Negative	Negative
3% (FD2)	Negative	Negative
Interaction (ST x FD)		
ST0FD0	Negative	Negative
ST0FD1	Negative	Negative
ST0FD2	Negative	Negative
ST1FD0	Negative	Negative
ST1FD1	Negative	Negative
ST1FD2	Negative	Negative
ST2FD0	Negative	Negative
ST2FD1	Negative	Negative
ST2FD2	Negative	Negative
ST3FD0	Negative	Negative
ST3FD1	Negative	Negative
ST3FD2	Negative	Negative
ST4FD0	Negative	Negative
ST4FD1	Negative	Negative
ST4FD2	Negative	Negative

Based on the research results in Table 2, *E. coli* and *Salmonella* sp. in all treatments are negative. These findings demonstrate that an edible coating treatment incorporating crab chitosan with DD of 79.52% and palm stearin effectively inhibits the growth of *E. coli* and *Salmonella* sp. This indicates that the antimicrobial effectiveness of chitosan is affected by its DD. Ke et al. [37] stated that chitosan has antimicrobial activity so that microbes cannot grow properly. Khubiev et al. [38] added that the antimicrobial activity contained in chitosan will have an impact on inhibiting microbial growth. Research conducted by Aprilianti et al. [39] found that casein–chitosan treatment also produced negative *Salmonella* sp. Ardean et al. [40] also noted that the antimicrobial effectiveness of chitosan depends on both its source and the degree of deacetylation.

## Conclusion

Chitosan–stearin edible coatings with different formula doses and STs consistently preserve the quality of fresh broiler meat during cold storage, with average values of water content ranging from 48.97% to 53.73%, water-holding capacity from 17.52% to 34.30%, cooking loss from 10.03% to 33.19%, pH levels from 4.93 to 5.53, protein content from 14.54% to 17.46%, fat content from 20.55% to 24.21%, and no detectable presence of *E. coli* and *Salmonella* sp.

## List of abbreviations

DD, degree of deacetylation; FD, formula dosage; ST, storage time.
